# Guest Editorial: Patient safety and errors in the application of medical radiation: our responsibility

**DOI:** 10.1120/jacmp.v11i2.3329

**Published:** 2010-04-16

**Authors:** Michael G. Herman

**Affiliations:** ^1^ American Association of Physicists in Medicine Professor and Chair, Division of Medical Physics Department of Radiation Oncology Mayo Clinic

I appreciate the opportunity to share with the JACMP readership some information gained from recent experiences and activities related to safety in the delivery of medical radiation. Medical Physics is the discipline of physics that brings the application of scientific methods to bear in medicine and biology. Medical physicists provide services that impact the quality and safety of patient care through direct clinical work, research and development and through education. We have each embarked on this career as a medical physicist, because we felt that our skills and knowledge could be used in a productive manner to benefit people in need of medical care. That care many times utilizes the complex technology we develop and bring to the clinic. We are directly responsible for the identification and implementation of improvements in patient safety for the medical use of radiation in imaging and radiation therapy.

Patients receive diagnostic and therapeutic radiation with the full intent and belief that they are receiving safe and effective care by a medical team to whom they entrust their lives. Recent articles in *The New York Times* as well as FDA initiatives and Congressional hearings have brought national attention to errors in the medical use of radiation, ranging from significantly increased doses received by patients having CT scans to patients receiving lethal excess doses of radiation during radiation therapy. Any error is one too many; we must pay attention to any event that causes harm to a patient.

The resulting national attention has allowed the associations that represent the medical team members, manufacturers, government agencies and others to express thoughts and experiences about the problems and solutions in the use of medical radiation. Even though the use of medical radiation impacts fewer patients than other areas of medicine, we are in the spotlight and have an opportunity to make landmark improvements in our domain of practice which could, in turn, be seen by the larger medical community as an example of better medicine.

To this end, the community representing the members of the medical team in radiation medicine have joined together to identify and address the common critical issues whose resolution could substantially improve safety and quality in the medical use of radiation.

The following issues were defined, in part, by the congressional testimony of almost every medical association at the hearing on medical radiation on Feb 26, 2010:

**Qualifications**. Individuals involved in the delivery of medical radiation for imaging and therapy must demonstrate competence through nationally recognized and consistent qualifications that guarantee proper education, clinical experience and specialty certification have been achieved.

ACR, ASTRO, ASRT, SROA and AAPM all strongly support the passage of the *Consistency, Accuracy, Responsibility and Excellence in Medical Imaging and Radiation Therapy (“CARE”) Act*. This legislation, and its subsequent implementation through the federal Department of Health and Human Services, would mandate the aforementioned consistency explicitly. Medical physicists remain actively involved in the work related to the eventual rollout of the CARE Act.

AAPM further urged that the legislation specifically require inclusion of all medical physicists involved in medical imaging and radiation therapy, so that the definition of qualified medical physicist was recognized uniformly and consistently, nationwide. This is relevant because, as written, CARE excludes individuals covered under the *Medicare Improvements for Patients and Providers Act* (MIPPA). MIPPA would review qualifications for team members in the accreditation process for advanced imaging (CT, PET, nuclear medicine, MRI) and could thus have very different requirements than CARE.

ASTRO further called for all radiation oncologists to be board‐certified and to participate in maintenance of certification.

**Accreditation**. Accreditation is a mechanism by which a practice is reviewed and evaluated to ensure quality, safety and consistency. While accreditation does ensure some standards and quality are achieved, the accreditation process for radiation oncology is not required, nor is it consistent between accrediting bodies. Variations in standards range from the accreditation process itself, to technical expectations and personnel qualifications.

ACR, ASTRO and AAPM all endorsed that improved and more consistent accreditation models be developed and implemented nationally. The goal is to define the model for national accreditation that would provide robust, safe and high‐quality care, without creating significant financial or other resource burdens. There will be different efforts and partnerships for imaging vs. therapeutic applications of radiation.

In radiation oncology, there are currently white papers being developed that will represent the basic quality assurance and technical items to be considered necessary to carry out safe and effective radiation therapy. These documents could be the structural basis for guiding a consistent set of accreditation standards. The white papers under development include IMRT, IGRT, SBRT and HDR.

In addition, a point was raised by Dr. Mike Hagan (who oversees the VA radiation oncology practices) that the accreditation and practice review process would benefit significantly from a technical review and audit process, akin to the work that the Radiologic Physics Center carries out for NCI and practices that participate in clinical trials. In the 1970s and early 1980s, federally funded centers for radiologic physics (CRPs) existed for the purpose of providing these services to any/all radiation oncology practices. The federal funding was eliminated in the mid‐1980s. The CRP model however could provide an independent audit and credentialing service that practices or accrediting bodies could utilize to objectively demonstrate technical competence in general and for specific procedures. AAPM has formed a committee to study the concept of reincarnating the CRP model to provide this technical service in a financially sustainable fashion.

In imaging, there are many efforts underway, primarily within the accrediting organizations for imaging modalities – ACR, IAC (intersociety accrediting commission), and now the Joint Commission (recently deemed for accreditation under MIPPA by CMS). Medical physicists have been and continue to be involved with these accrediting bodies to help bring consistency to the medical physics and technical components. This work is essential as additional bodies gain status to accredit imaging practices. Medical physicists must continue to reach out, in an organized and consistent manner, to guide the accreditation process for robust and safe practice.

In either case, accreditation based on community consensus practice guidance that recognizes qualified individuals, establishes minimum staffing levels and has additional accreditation requirements for advanced highly‐specialized procedures is required and, to be effective, should be linked to payment for services. Whether the more prescriptive model such as MQSA or a more flexible model such as MIPPA should be mandated is a significant discussion point. In the MQSA model, the process, staffing and qualifications are defined by mandate and are consistent nationwide. In the MIPPA environment, each accrediting body has significant latitude in determining required process, staffing and personnel qualifications to meet their accrediting standards.

**National Reporting/Tracking**. There is no consistent mechanism for reporting potential or actual adverse events in the medical use of radiation. Some are reported through NRC, some through FDA, some at various state agencies or within medical center administration. Definitions and thresholds are inconsistent and confusing to the community. This was one of the primary topics reflected in Congressman Markey's questions to the witnesses following the Feb 26 hearings. ACR, ASTRO, ASRT and AAPM have all endorsed an action that would create a unified, consistent and national event reporting mechanism/system that could be used by all stakeholders, and would provide essential data on medical errors to perform trend analysis and assessment, and be used to inform the community and make improvements. It will require cooperative work with federal and state regulatory agencies to coordinate this effort. One significant need is to develop a consistent terminology/nomenclature to be used in a national reporting system. Medical physicists are actively engaged in developing such an infrastructure. In addition, the medical community is in active discussion with federal agencies to coordinate a roundtable discussion on centralized reporting.

**Manufacturers and the FDA Process**. The FDA premarket approval and postmarket monitoring systems have limitations in terms of effectively reviewing all safety and efficacy aspects of devices that use medical radiation. This leads to devices being brought to market without a complete understanding of limitations and with some remaining safety concerns. There is no well‐defined mechanism for taking action from findings derived from postmarket monitoring. Active discussions with FDA and other organizations to help define a more quantitative and robust premarket approval process are already underway. Numerous federal listening sessions have been conducted over the past 12 months on this topic and medical physicists have participated in all of them. It is also possible that technical assessments performed by expert entities that are independent of manufacturer and regulator could provide objective, quantitative and relevant guidance to the clearance and monitoring process.

**Improving Safety**. In addition to actions mentioned above, there are specific short‐term actions to bring awareness to the community at large, both in imaging and therapy. A CT summit, being held at the end of April 2010, will provide specific guidance for all CT team members to the understanding of CT technique, calibration and monitoring to ensure that the highest quality images are achieved with the smallest possible dose. A Safety in Radiation Therapy – A Call to Action meeting is being conducted at the end of June, 2010. It will focus on the inherent problems associated with recent medical errors and on the culture of safety necessary to deliver high‐quality radiation therapy with an absolute minimum number of adverse events. There will continue to be error prevention and reduction educational workshops and sessions at many venues for years to come.

**Our Responsibility**. Each of us has the responsibility to do the very best for every patient service we provide. This is true in R&D, education and in the clinic. We should read and follow national consensus (and regulatory) guidance on all procedures. Pressure to perform more efficiently or to increase volume must be secondary to patient safety and high‐quality care. This is absolute. Those of us involved in the research and development process must build safety mechanisms into the technology we develop so that clinical team members have reduced potential for committing errors. Educators must embrace and teach a culture of safety. We do what we do to the benefit of every patient.

